# Commentary: Cancer-testis antigen lactate dehydrogenase C4 as a novel biomarker of male infertility and cancer

**DOI:** 10.3389/fonc.2023.1115620

**Published:** 2023-02-06

**Authors:** A. Naik, J. Decock

**Affiliations:** ^1^ Translational Cancer and Immunity Center (TCIC), Qatar Biomedical Research Institute (QBRI), Hamad Bin Khalifa University (HBKU), Qatar Foundation (QF), Doha, Qatar; ^2^ College of Health and Life Sciences (CHLS), Hamad Bin Khalifa University (HBKU), Qatar Foundation (QF), Doha, Qatar

**Keywords:** LDHC, lactate dehydrogenase C, cancer biomarker, therapeutic target, cancer testis antigen

We read with great interest the recently published review by Jing W et al. (1) on the relevance of the cancer testis antigen (CTA) Lactate Dehydrogenase C (LDHC) in male infertility and cancer. In their review, the authors discussed the mechanisms by which LDHC regulates sperm function and supports tumorigenesis. Overall, its best characterized function is the regulation of glycolysis whereby LDHC catalyzes the interconversion of pyruvate and NADH to lactate and NAD+ resulting in rapid ATP production. The loss of LDHC expression and concomitant reduction in ATP production greatly compromises spermatozoa motility and cAMP/PKA-mediated sperm capacitation preceding fertilization. In cancer, the enzymatic activity of LDHC plays a key role in metabolic reprogramming of cancer cells from oxidative phosphorylation to aerobic glycolysis to support the increasing energy demands for rapid growth as described by Jing W et al. However, the pro-tumorigenic effects of this metabolic switch extend beyond cancer cell energetics. We have previously reported on the role of cancer cell metabolic reprogramming in shaping an immunosuppressive environment whereby metabolic competition for glucose directly impairs immune cell functionality and lactic acidosis further inhibits the proliferation and activity of infiltrating cytotoxic immune cells (2). These findings suggest that cancer cell metabolism and immunosuppression are closely interconnected, however, it remains to be determined whether LDHC plays a role at this interface.

High expression of LDHC has been associated with poor clinical outcome in the majority of cancer types, and favorable prognosis in a select few cancers such as head and neck squamous cell carcinoma (HNSC) and cervical squamous cell carcinoma and endocervical adenocarcinoma (CESC). However, very little is known about the molecular mechanisms underlying the oncogenic role of LDHC. As summarized by Jing W and colleagues, overexpression of LDHC in renal cell and lung cancer cell lines and a lung cancer xenograft mouse model greatly enhances cellular motility through regulation of epithelial-to-mesenchymal transition (EMT) and expression of matrix metalloproteinases (3, 4). In our recent work, we demonstrated that LDHC is involved in the regulation of the DNA damage response (DDR) and long-term survival capabilities of breast cancer cells (5). We found that silencing of LDHC induces the formation of giant cells with increased nuclear aberrations and microtubule destabilization. Molecularly, we observed that downregulation of LDHC dysregulates various cell cycle checkpoints, resulting in the accumulation of DNA damage and driving cancer cells towards mitotic catastrophe and ultimately cell death. In addition, we identified cell line-dependent changes in the expression of single strand and double strand DNA damage sensors such as ATM, ATR, and DNA-PKC. Moreover, combination treatment of LDHC silencing with DDR drugs such as cisplatin and olaparib significantly increased DNA damage and reduced cancer cell survival. Our findings strongly support the therapeutic potential of targeting LDHC to directly affect tumor cell survival or to improve treatment response to existing anti-cancer drugs. Furthermore, although LDHC expression is generally thought to be restricted to the cytosol of somatic cells, our observations suggest that LDHC may potentially translocate to the nucleus during the DNA damage response.

While our study was the first report to show an involvement of LDHC in the DNA damage response, these findings are supported by the notion of other CTAs being implicated in similar biological processes. For instance, HORMA domain containing protein 1 (HORMAD1) impairs the function of the DNA mismatch repair machinery and was shown to promote treatment resistance to docetaxel by enhancing DNA damage tolerance (6). ATPase family AAA domain containing 2 (ATAD2) was found to modulate the expression of DNA damage response and repair factors, and was required for the resolution of DNA damage foci and activation of homologous recombination. Furthermore, silencing of *ATAD2* in triple negative breast cancer cells increased the treatment efficacy of the DNA damaging drug carboplatin (7). In addition, several CTAs have been implicated in regulating genomic instability such as Synaptonemal Complex Protein 3 (SYCP3), SPO11 Initiator Of Meiotic Double Stranded Breaks (SPO11) and members of the synovial sarcoma, X-breakpoint (SSX2) family (8–10). Moreover, in analogy with the widely studied cancer testis antigens NY-ESO-1, PRAME and MAGE-A3, we previously identified 2 major immunogenic HLA-A2* restricted peptides within LDHC that can induce cytotoxic T cell responses (11). As highlighted by Jing W and colleagues, the study by Triozzi et al. further suggests that T cell LDHC expression could be used as a predictive biomarker for PD-1 blockade response (12).

To conclude, the restricted expression pattern of LDHC together with its immunogenic nature and oncogenic behavior make it an ideal candidate for therapeutic intervention. We believe that LDHC holds great therapeutic potential to directly target cancer cells, sensitize cancer cells to DDR drugs, and induce specific anti-tumor immune responses. Furthermore, LDHC may exhibit clinical value as a prognostic and predictive biomarker to facilitate patient stratification. The current studies focusing on LDHC in cancer, although limited in number, show great promise and warrant further investigation.

## Author contributions

JD and AN contributed to writing the manuscript. All authors read and approved the submitted version.
